# Key Targets for Multi-Target Ligands Designed to Combat Neurodegeneration

**DOI:** 10.3389/fnins.2016.00375

**Published:** 2016-08-22

**Authors:** Rona R. Ramsay, Magdalena Majekova, Milagros Medina, Massimo Valoti

**Affiliations:** ^1^Biomedical Sciences Research Complex, University of St. AndrewsSt. Andrews, UK; ^2^Department of Biochemical Pharmacology, Institute of Experimental Pharmacology and Toxicology, Slovak Academy of SciencesBratislava, Slovakia; ^3^Departamento de Bioquímica y Biología Molecular y Celular, Facultad de Ciencias and BIFI, Universidad de ZaragozaZaragoza, Spain; ^4^Dipartimento di Scienze della Vita, Università degli Studi di SienaSiena, Italy

**Keywords:** multi target designed ligands, mitochondria, oxidative stress, monoamine oxidase, cytochrome P450, neurodegeneration

## Abstract

**HIGHLIGHTS**

- Compounds that interact with multiple targets but minimally with the cytochrome P450 system (CYP) address the many factors leading to neurodegeneration.
- Acetyl- and Butyryl-cholineEsterases (AChE, BChE) and Monoamine Oxidases A/B (MAO A, MAO B) are targets for Multi-Target Designed Ligands (MTDL).
- ASS234 is an irreversible inhibitor of MAO A >MAO B and has micromolar potency against the cholinesterases.
- ASS234 is a poor CYP substrate in human liver, yielding the depropargylated metabolite.
- SMe1EC2, a stobadine derivative, showed high radical scavenging property, *in vitro* and *in vivo* giving protection in head trauma and diabetic damage of endothelium.
- Control of mitochondrial function and morphology by manipulating fission and fusion is emerging as a target area for therapeutic strategies to decrease the pathological outcome of neurodegenerative diseases.

Growing evidence supports the view that neurodegenerative diseases have multiple and common mechanisms in their aetiologies. These multifactorial aspects have changed the broadly common assumption that selective drugs are superior to “dirty drugs” for use in therapy. This drives the research in studies of novel compounds that might have multiple action mechanisms. In neurodegeneration, loss of neuronal signaling is a major cause of the symptoms, so preservation of neurotransmitters by inhibiting the breakdown enzymes is a first approach. Acetylcholinesterase (AChE) inhibitors are the drugs preferentially used in AD and that one of these, rivastigmine, is licensed also for PD. Several studies have shown that monoamine oxidase (MAO) B, located mainly in glial cells, increases with age and is elevated in Alzheimer (AD) and Parkinson's Disease's (PD). Deprenyl, a MAO B inhibitor, significantly delays the initiation of levodopa treatment in PD patients. These indications underline that AChE and MAO are considered a necessary part of multi-target designed ligands (MTDL). However, both of these targets are simply symptomatic treatment so if new drugs are to prevent degeneration rather than compensate for loss of neurotransmitters, then oxidative stress and mitochondrial events must also be targeted. MAO inhibitors can protect neurons from apoptosis by mechanisms unrelated to enzyme inhibition. Understanding the involvement of MAO and other proteins in the induction and regulation of the apoptosis in mitochondria will aid progress toward strategies to prevent the loss of neurons. In general, the oxidative stress observed both in PD and AD indicate that antioxidant properties are a desirable part of MTDL molecules. After two or more properties are incorporated into one molecule, the passage from a lead compound to a therapeutic tool is strictly linked to its pharmacokinetic and toxicity. In this context the interaction of any new molecules with cytochrome P450 and other xenobiotic metabolic processes is a crucial point. The present review covers the biochemistry of enzymes targeted in the design of drugs against neurodegeneration and the cytochrome P450-dependent metabolism of MTDLs.

## Introduction

Neurodegeneration is a complex process that can arise from many different defects or insults. In the last five years at least 80 reviews with “neurodegeneration” in the title have appeared, each covering different aspects of the processes involved. These include protein aggregation, mitochondrial movement, and function, dysregulation of microRNA, iron accumulation, inflammation, defects in proteins such as sirtuins or tau, dysregulation of protein trafficking or breakdown, and oxidative stress (Donmez, 2012; Gascon and Gao, 2012; Schipper, 2012; Sheng and Cai, 2012; Costanzo and Zurzolo, 2013; Butterfield et al., 2014; Moussaud et al., 2014; Rao et al., 2014; Wang X. et al., 2014; Witte et al., 2014; Goedert, 2015; Sankowski et al., 2015). With such complexity, it has proved difficult to identify biomarkers to quantify progression and targets to block to prevent the degeneration. The most obvious physiological symptoms are the loss of neurons in Alzheimer's Disease (AD) and Parkinson's Disease (PD) with the consequently lower neurotransmitter levels, and the formation of protein aggregates in all forms of neurodegeneration. These observations provided the primary targets to date, namely enzymes catalyzing neurotransmitter breakdown (cholinesterases, ChE; monoamine oxidases, MAO; catechol-O-methyltransferase, COMT), prevention of production of amyloid beta (Aβ) by beta-secretase, of protein aggregation, and of oxidative damage known to stress cells to the point of apoptosis (Guzior et al., 2015; Swomley and Butterfield, 2015). Intervention in the potentially damaging outcomes of oxidation stress either by means of upstream (prevention of free radical generation) or downstream (free radical scavenging) antioxidant pathways helps preserve neurons and slow neurodegeneration (Uttara et al., 2009).

Related to oxidative damage and because of their role in the regulation of apoptosis, mitochondria are a current active area of investigation both for generation of reactive oxygen species (ROS) or inefficient energy production that limits defenses against ROS. Recent advances focus on mitochondrial movement, fusion, and fission and interactions with the cytosol (including specific proteins related to neurodegeneration such as the Bax/Bid family and sirtuins) (Eckert et al., 2012; Johri and Beal, 2012). Mitochondria play a central role in the oxidative metabolism of nutrients and ATP synthesis. They also contribute to intracellular second messenger homeostasis (Ca^2+^ and ROS), and are determinant for both cell survival and apoptotic cell coordination (Waagepetersen et al., 2003; Mandemakers et al., 2007; Nunnari and Suomalainen, 2012; Bernardi et al., 2015). Mitochondrial dysfunction is frequently proposed to be involved in neurodegenerative pathogenesis, including PD and AD (Mandemakers et al., 2007; Moreira et al., 2010; Correia et al., 2012a,b; Perier et al., 2012; Perier and Vila, 2012). With their high energy demands neurons are particularly dependent on mitochondrial ATP generation, and are thus intolerant of mitochondrial dysfunction (Lezi and Swerdlow, 2012). This makes the understanding of the mitochondrial mechanisms underlying these pathologies critical for designing more effective strategies to halt or delay disease progression (Correia et al., 2010a,b). An alternative strategy to preserving levels of neurotransmitters by inhibiting breakdown is the pharmacological stimulation of the post-synaptic receptors in the remaining neurons. Most receptors are G-protein coupled receptors (http://www.guidetopharmacology.org/), an area of fast recent progress with the determination of receptor structures, such as the muscarinic acetylcholine receptors (Thal et al., 2016), the availability of cloned receptors for pharmacology and compound screening (Katritch et al., 2013; Melancon et al., 2013), and new methods for assessing the complex function of the receptors (van Unen et al., 2015). Here too, multiple targets are attractive: for example, first-in-class dual M1/M4 agonists now in preclinical development (http://www.heptares.com/pipeline/). Antagonists to histamine receptors are also interesting to prevent the inflammation also thought to contribute to neurodegeneration (Vohora and Bhowmik, 2012; Walter and Stark, 2012). However, a meta-analysis of placebo-controlled trials for H3 receptor antagonists did not find significant effects on cognition (Kubo et al., 2015). Receptors will not be further mentioned in this article because we focus on intracellular targets.

It is apparent from the above outline that the primary causes of neurodegeneration are not easily defined, and will almost certainly be due to highly individual combinations of factors. This has led to the search for novel compounds that will interact with multiple targets, and have antioxidant properties as part of the desired pharmacologic profile. For the future there will be a need for various combinations of multi-target designed ligands (MTDL) to meet the needs of each individual combination of defects. In this article, we shall describe the background to *in vitro* assessment of compounds to combat neurodegeneration, considering the current targets either for symptomatic treatment (AChE and MAO) or to prevent or reverse deterioration (anti-oxidants or mitochondrial function), and giving examples of compounds from our own work conducted in collaborations facilitated by COST Action CM1103 “Structure-based drug design for diagnosis and treatment of neurological diseases: dissecting and modulating complex function in the monoaminergic systems of the brain.”

Screening techniques highlight that many enzymes and receptors interact with a given chemical. This is clear in off-target data-mining (Nikolic et al., 2015; Hughes et al., 2016) and in high throughput screens (Sipes et al., 2013). In the latter project aimed at building a resource of biological pathways of toxicity for various types of chemicals, 976 compounds known as pharmaceuticals, food additives or pesticides were tested for inhibition or activation of enzymes and for binding to monoaminergic transporters and receptors. The most common sub-micromolar interactions were with the cytochrome P450 (CYP) family, transporters, the mitochondrial translocator (benzodiazepine–binding) protein, the dopamine and serotonin reuptake carriers, and the aminergic G-protein coupled receptors, and MAO was also in the top 20 most promiscuous proteins. These results indicate the promise of MTDL for cholinesterase (lower on that list) and MAOs or to include receptor agonism or antagonism into one molecule is not without the drawback of also finding off-target activity. In particular, any effect on the metabolic CYP enzymes must be carefully appraised.

After the identification of the target, be it receptor or enzyme, a variety of empirical and/or *in silico* studies are conducted in order to vary the structure to increase the pharmacological effects of the new compounds. However, good *in vitro* activity may not correspond to a therapeutic effect, unless the molecule also possesses high bioavailability and low toxicity. This means that the new compounds must have good pharmacokinetic properties. The investigation on absorption, distribution, metabolism and excretion properties and toxicological profiling (ADME/Tox) have become an essential step in early drug discovery that has demonstrated a high impact on the successful progression of drug candidates. Growing knowledge of the key roles that pharmacokinetics and drug metabolism play as determinants of *in vivo* drug action, has led many researchers, drug companies and regulatory agencies to include examination of pharmacokinetics and drug metabolism properties as part of their process in the selection of drug candidates. In this context, the role of the CYP isoenzymes is outlined, since it represents a major source of variability toward pharmacokinetics and pharmacological responses in this phase.

In this review we consider the biochemistry of some of the key pharmacological targets of MTDL, giving selected examples from our own expertise. The traditional key targets in Alzheimer's Disease (AD), the ChEs and MAOs, are described first, then the new and diverse potential targets in mitochondrial function for cell survival, followed by an example of targeting the oxidative stress that is seen in a variety of degenerative conditions. Lastly in this overview of metabolic aspects of drug design, the action of the CYP isoezymes, important for effectiveness of all drugs *in vivo*, on MTDL is described.

## Addressing the pathology of neurodegeneration: the targets considered here

Four of the five drugs ever approved to treat symptoms of memory loss and confusion in AD patients are cholinesterase inhibitors. The cholinergic hypothesis of AD posits that the cognitive and behavioral dysfunctions of AD result from deficits in acetylcholine neurotransmission. These early symptoms can be ameliorated by inhibiting the cholinesterases to prolong the presence of acetylcholine in the synapse. However, cholinesterase activities have also been reported correlate with the density of amyloid plaque deposition in the AD brain (Arendt et al., 1992). The mechanism by which AChE and to a lesser extent BChE facilitate plaque deposition is still being investigated (Hou et al., 2014).

The other catabolic enzyme that is inhibited to maintain decreasing neurotransmission is MAO, located on the cytosolic face of the mitochondrial outer membrane where it is attached by a single membrane-spanning helix. To be metabolized by MAO, monoamine neurotransmitters must be taken up into the cells. The two forms, MAO A and MAO B, are co-located in liver mitochondria, but otherwise have very different expression patterns. MAO A is the major form in the intestine and placenta, MAO B in platelets. In the brain, MAO B is expressed in the glia and in serotonergic neurons, whereas MAO A predominates in all other neurons.

Mitochondria produce the majority of energy in all type of cells but particularly in neurons where the energy demand for neurotransmission is high. Deficits in mitochondrial function (i.e., increased oxidative stress, decreased efficiency of the respiratory chain, apoptosis dysfunctions, deregulation of fusion and fission processes) have been found in all neurodegenerative conditions. Understanding the mechanisms underlying these pathologies is critical to designing more effective strategies to halt or delay disease progression (Correia et al., 2010a, 2012a). Each of the mitochondrial functions is closely related to the others and alteration in any of them might develop neurodegeneration, making difficult to discriminate which changes are more critical (Haddad and Nakamura, 2015). Abnormal morphology, altered dynamics, and biochemical dysfunction of mitochondria are usually observed, being often systemic rather than brain-limited (Lezi and Swerdlow, 2012).

Mitochondrial respiratory capacity and efficient ATP production are vital for neuronal survival. In most neurodegenerative conditions mutations accumulate in mitochondrial DNA (encoding 13 proteins essential for respiratory chain function), the enzymatic activity of respiratory chain enzymes is altered and oxidative stress usually increases (Goldberg et al., 2002). Such dysfunctions arise not only as consequence of mutations in mitochondrial DNA but can also be due to mutations in nuclear DNA encoding for proteins either imported to or interacting with the mitochondria. Changes in the mitochondrial membrane potential and the increased reactive oxygen species (ROS) associated with electron transport chain dysfunction have been strongly linked to reduced cell viability (Bird et al., 2014). Although ROS formation is a natural by-product of mitochondrial respiration, overproduction is indicative of cell stress (Murphy, 2009). Antioxidant therapy has therefore long been sought to combat aging as well as neurodegeneration.

Antioxidant compounds can either react with radicals to prevent damage to biological molecules (proteins, lipids or DNA) or can complex metal ions to decrease generation of ROS. Iron ions are the well-established target in AD, playing a key catalytic role in the Fenton reaction (reviewed in Unzeta et al., 2016). Knock out studies have established that loss of Amyloid Precursor Protein (APP) or tau (both AD-linked proteins) results in iron accumulation in the brain. Iron is bound to ferritin, a protein that increases with age and in AD (Bartzokis and Tishler, 2000). Iron is found also in plaques (Meadowcroft et al., 2009). Iron-chelation capability is part of the action spectrum of rasagiline used for treatment of PD (Weinreb et al., 2016) and a highly desirable addition to future MTDL compounds for prevention of neurodegeneration.

Lastly, decreasing the generation of aberrant proteins, preventing their aggregation, and blocking down-stream events are developing targets. The prevention of production of amyloid beta (Aβ) by inhibition of the beta-secretases already has led to candidate small molecule compounds in clinical trials (Yan and Vassar, 2014; Yan et al., 2016). The acceleration of Aβ aggregation by the peripheral site of AChE has long been recognized (Inestrosa et al., 1996; Reyes et al., 1997) and is an important component of effective AChE inhibitors designed to combat AD (Bartolini et al., 2003; Anand and Singh, 2013; Bolea et al., 2013; Bautista-Aguilera et al., 2014a; Hebda et al., 2016). Deleterious intracellular effects of Aβ are also recognized, such as the consequences of Aβ binding to a 17-β-hydroxysteroid dehydrogenase known as Amyloid Binding Alcohol Dehydrogenase (ABAD), a tetrameric mitochondrial enzyme that catalyzes the oxidation of steroids. ABAD is decreased in AD (Lustbader et al., 2004) and missense mutations in its gene (HSD17B10) result in alteration of mitochondria morphology and neurodegeneration in infancy (Yang et al., 2014). The ABAD-Aβ interaction is associated with up-regulation of endophilin, a protein important for membrane-shaping in processes such as synaptic vesicle formation which might contribute to neuronal sensitivity to ABAD-Aβ complex formation inside neuronal mitochondria (Borger et al., 2013). Drug discovery to prevent the ABAD-Aβ interaction, begun with brain-permeant peptides (Borger et al., 2013), is now moving to small molecules (Valaasani et al., 2014; Benek et al., 2015; Hroch et al., 2015) that will provide information for future incorporation into multi-target compounds.

The aim of MTDL design is to combine features that can interact with two or more of the desired targets (Csermely et al., 2005; Geldenhuys et al., 2011; Hughes et al., 2016). This expands the biological screening required at the early stages for hit discovery and lead optimization. With structures of most targets available, *in silico* screening is a useful tool for examining large chemical databases (Hughes et al., 2016; Nikolic et al., 2016). Combining known drugs for each target into one molecule has also produced promising compounds by incorporating elements of proven inhibitors for each target into new multi-potent molecules (Bolognesi et al., 2007; Piazzi et al., 2008; Zhu et al., 2009; Kupershmidt et al., 2012; Luo et al., 2013; Sun et al., 2014; Bautista-Aguilera et al., 2014b; Wang L. et al., 2014; Pisani et al., 2016; Weichert et al., 2016; Xie et al., 2016). One example that progressed to clinical trials against AD is ladostigil, designed to inhibit MAOs and ChEs but also incorporating potent anti-apoptotic and neuroprotective activities (Weinreb et al., 2012; Youdim, 2013). The next sections in this review will consider other examples of MTDL in the context of some of these targets of interest for AD drugs.

## Neurotransmitter degrading enzymes

### Enzyme inhibitors—pharmacological characterization

The development of novel drugs that target enzymes requires an understanding of enzyme mechanism and is deeply informed by detailed knowledge of the protein structure. Understanding how enzymes (or indeed receptors) work is vital for medicinal chemists aiming to design new drugs (Walsh, 2013). In the very first stage of evaluation of new compounds in a biological system, the medicinal chemistry shortcut of IC_50_ measurement is an invaluable tool for comparisons of series of derivatives on a given scaffold and provides useful information for determining a hit or for choice of a lead compound. It is a measurement that can be used for both simple and complex biological systems but it is important to recognize that the meaning of IC_50_ (as opposed to its definition as 50% inhibition of a measured parameter) changes according to the system and the assay conditions. In the context of measurement of a single enzyme activity, IC_50_ is not affinity for a target but simply the concentration of the compound that inhibits the activity by 50% under the specific conditions used. For more informative data on enzyme reversible inhibitors, the K_i_ (the inhibition constant independent of the substrate concentration used) and the mechanism of inhibition should be determined. For irreversible inhibitors, the rate of inactivation and the concentration dependence are needed (McDonald and Tipton, 2012). It should be recognized that IC_50_ values for reversible and irreversible inhibitors are not directly comparable because of the time element. The initial reversible binding of an inactivating inhibitor can only be compared with reversible inhibitors (or indeed binding constants from docking) if initial rates are measured in an assay where the enzyme is added last to a mixture of substrate and inhibitor.

When comparing alternate targets, care is needed to use conditions for each target that will allow comparison. Selective inhibition of MAO A and MAO B is often desired, but they have different K_M_ values for their common substrates (the concentration of substrate required to give half the maximum velocity), so are saturated to different degrees at any one concentration. For example, purified human MAO A activity reaches 50% of its maximum at 0.15 mM kynuramine, whereas MAO B reaches 50% of maximum rate with only 0.08 mM kynuramine. With reversible inhibitors,

E + I ↔ EI but during steady state measurement, when E + S ↔ ES → E + P, the concentration of free enzyme (E) available to bind inhibitor is not the total enzyme added but rather a fraction of the total that depends on the substrate concentration used and the relative values of the rate constants. In the steady-state where ES is constant, MAO A assayed with 0.1 mM kynuramine has 60% of free enzyme but MAO B has only 44% available for inhibitor binding. For an inhibitor of both with the same K_i_ of 0.01 mM, the IC_50_ would be measured as 17 μM for MAO A but 22.5 μM for MAO B despite the fact that the inhibitor bound equally well to both enzymes. Simply using an assay with fixed substrate concentration without taking into account the different K_M_ values would therefore introduce a 30% bias to the selectivity.

The mechanism of the enzyme can also influence IC_50_ values. This is seen in the kinetic analysis of MAO B where it is clear that there are two forms of the enzyme that can bind the ligand (substrate or inhibitor), namely, the oxidized or the reduced forms, and that these two forms bind ligands with different affinities. Since different substrates give different proportions of these forms during steady-state catalysis, different Ki values for an inhibitor can be obtained from different substrates. Overall, care must be exercised in choice of substrate and of assay conditions to obtain reliable IC_50_ values, but only kinetic constants can be considered meaningful (McDonald et al., 2010; Ramsay et al., 2011). Slow and tight binding inhibitors also require special analysis (Morrison, 1969).

For irreversible inhibition, a time course of the development of the inactive enzyme is essential. The best compounds for specific irreversible inhibition *in vivo* are mechanism-based inhibitors, making use of the catalytic specificity of the target itself. However, sometimes even mechanism-based activation to a reactive product can be catalyzed by more than one enzyme, as seen for the MAO inhibitor tranylcypromine that irreversibly modifies the flavin in MAO after single electron oxidation (Silverman, 1983; Bonivento et al., 2010). Tranylcypromine was recently found to modify also the flavin in the epigenetic histone demethylase enzyme LSD1 (Schmidt and McCafferty, 2007; Binda et al., 2010). For medicinal chemistry screening, irreversible inhibition can be detected as a decrease in the IC_50_ value after 30 min preincubation compared to no preincubation. For example, for MAO B the IC50 for tranylcypromine without preincubation is 4 μM but if preincubated with the enzyme for 30 min before substrate is added, the IC_50_ is 0.074 μM (Malcomson et al., 2015). Proper characterization of mechanism-based inactivation requires measurement of the rate of production of inactive enzyme over time with several inhibitor concentrations to obtain K_I_ and k_inact_ (Kitz and Wilson, 1962).

Catalysis consists of both binding and kinetics steps. Theoretical screening measuring the sum of the optimal interactions between a compound and a target addresses only binding (and that with limitations depending on the restrictions placed on molecular dynamics). As a result, enzyme IC_50_ values are frequently not in accord with computed binding constants. Although K_i_ and K_D_ can be numerically the same if measurements are made in a simple Michaelis-Menten system, they never have the same meaning. Nonetheless, theoretical screening is a useful tool, particularly for large compound libraries and to facilitate repurposing of existing drugs used for other clinical targets (Hughes et al., 2016; Nikolic et al., 2016).

### Cholinesterases (AchE, BchE)

#### AChE/BChE location, structure, activity, redundancy

**Figure 1:**
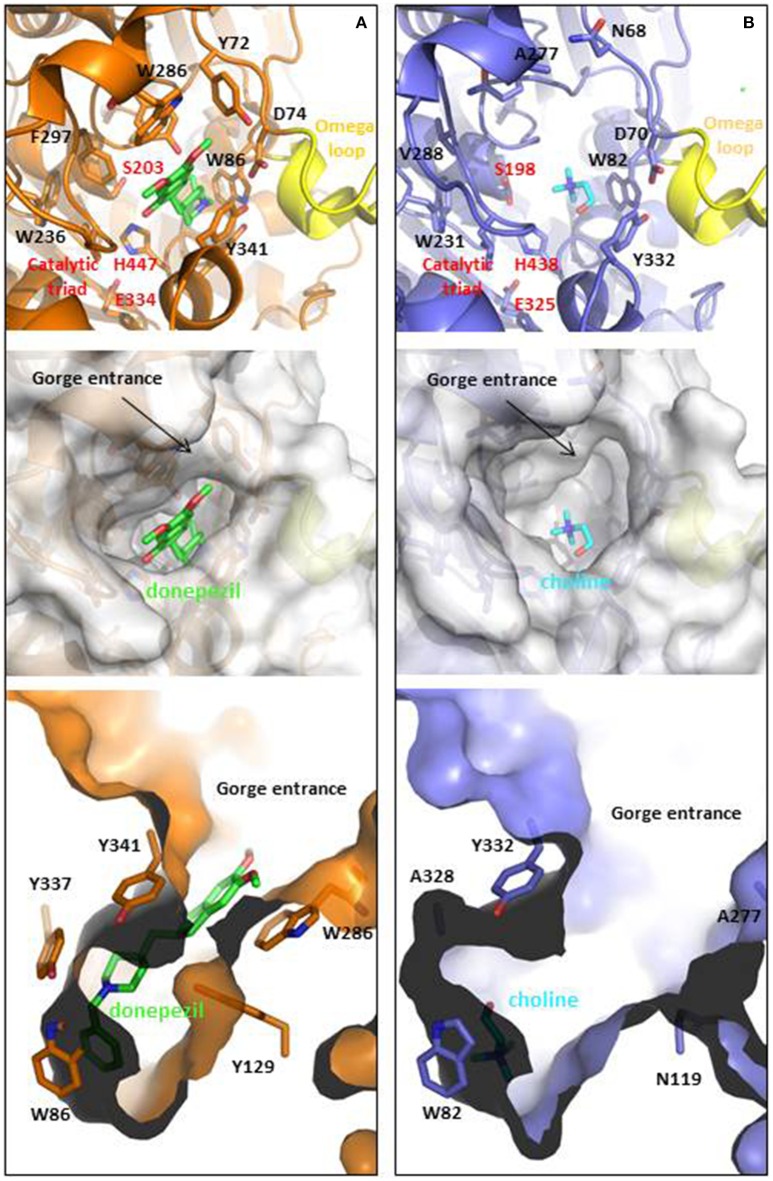
**Ligand binding cavities of (A) AChE and (B) BChE**. AChE (shown in orange) is in complex with donepezil (shown in CPK colored sticks with carbons in green, PDB ID: 4EY7), while BChE (shown metal blue) is in complex with choline (shown in CPK colored sticks with carbons in light blue, PDB ID: 1P0M). The top panels show the cartoon representations with detail in sticks of relevant residues involved at the gorge entrances, the PAS regions or the catalytic triads (labeled in red), as well as the omega loops colored in yellow. Middle and lower panels show top and lateral views of the ligand binding cavities. The entrance loops are highlighted in pink and yellow respectively. PDB files were obtained from the protein databank and figures were produced using the PyMol software (PyMOL, http://www.pymol.org).

AChE is located at neuromuscular junctions and in the central nervous system on the outside of the post-synaptic cell membranes, mainly in a tetrameric form. A Ser-His-Glu catalytic triad in the active site catalyzes the hydrolysis, and anionic and hydrophobic groups in the peripheral anionic site (PAS) contribute to binding a wide range of chemical structures (Figure 1). The drug, donepezil, spans both sites with aromatic stacking contributing to the nanomolar binding affinity, as shown in the crystal structure of the human AChE (Cheung et al., 2012). The PAS has a function in allosteric modulation of AChE activity and in increasing amyloid (Inestrosa et al., 2008; Hou et al., 2014).

BChE may also have a role in amyloid plaque formation (Darvesh et al., 2012). It is found mainly in plasma as a soluble monomer secreted by glial cells (Greig et al., 2002). Although the two enzymes share 65% homology and a similar hydrophobic active site structure, they have different specificities in part due to two aromatic residues (Phe295 and Phe297) that constrict the 20 Å long gorge in AChE (Greig et al., 2002; Nicolet et al., 2003). The K_M_ values and turnover numbers with acetylcholine are 0.1 mM and 6500 s^−1^ for AChE and 0.15 mM and 1433 s^−1^ for BChE (http://www.brenda-enzymes.org). In the normal brain where AChE is localize on the post synaptic membrane it was estimated that 90% of acetylcholine hydrolysis is catalyzed by AChE (Greig et al., 2002). However, BChE is plentiful and secreted by the glial cells so that if AChE is inhibited or is defective as in AChE-knock-out mice, the hydrolysis can be catalyzed by BChE (Mesulam et al., 2002). Thus, in current efforts to design multitarget drugs, reversible inhibition of both AChE and BChE is considered desirable.

#### Cholinesterase assay and inhibitors

Both AChE and BChE can be assayed using acetylthiocholine, but butyrylthiocholine is selective for BChE. The enzymes hydrolyse acetylthiocholine to acetate and thiocholine. Thiocholine reacts with Ellman's reagent (DTNB) to form a mixed dithiol, liberating 5-thio-2-nitrobenzoate that absorbs at 412 nm. The molar absorption coefficient is 14,150 M^−1^ cm^−1^; (Riddles et al., 1979) but it can vary slightly with salt concentration, pH, and temperature (Ellman et al., 1961; Eyer et al., 2003). The K_M_ for acetylthiocholine (0.025–0.05 mM) is similar for both enzymes although the rate with BChE is slower.

Common drugs inhibiting AChE and BChE are donepezil and tacrine (see Table 1) (Camps et al., 2008; Esteban et al., 2014; Wang L. et al., 2014). Carbamates are also reversible inhibitors (e.g., rivastigmine), coumarins, and several natural compounds have also been investigated. Harmine, an endogenous compound from the breakdown of tryptophan is also an inhibitor (He et al., 2015). In the last 5 years most inhibitor development has focused on maintaining a relatively equal inhibitory activity against AChE and BChE with IC_50_ values below μmolar concentrations in a compound that also acts on other targets such as MAO (see below), antioxidant, metal chelation, and preventing protein aggregation (for reviews see: León et al., 2013; Swomley and Butterfield, 2015). Many groups have synthesized and tested a variety of combinations. Here, we consider in detail ASS234, an example from our own work. ASS234 (Table 1) with potency similar to tacrine is almost equipotent on human AChE and BChE. ASS234 also has antioxidant properties, inhibits Aβ aggregation, and decreases Aβ-induced apoptosis in cellular studies (Bolea et al., 2013).

**Table 1:** **AChE and MAO inhibitors and the inhibitory activity of MTDL**.

**Structure**	**Compound**	**AChE (μM)**	**BChE (μM)**	**MAO A (nM)**	**MAO B (nM)**	**References**
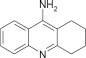	Tacrine	0.205^1^	0.044^1^	–	–	^1^Camps et al., 2008
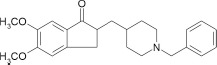	Donepezil	0.012^1^ 0.011^2^	7.3^1^ 6.22^2^	*850000^3^* *Rat*	*15000^3^* *Rat*	^1^Camps et al., 2008; ^2^Esteban et al., 2014; ^3^Wang L. et al., 2014
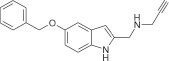	PF1901N	>100^2^	>100^2^	790^2^	11^2^	^2^Esteban et al., 2014
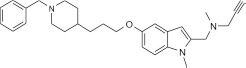	ASS234	*0.35^4^* *Eel* 0.81^2^	*0.46^4^* *Eel* 1.82^2^	5.24^4^ 0.17^2^	43350^4^ 15830^2^	^2^Esteban et al., 2014; ^4^Bolea et al., 2011
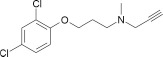	Clorgyline	–	–	0.42^2^	10660^2^	^2^Esteban et al., 2014
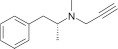	L-Deprenyl	–	–	630^2^	3.0^2^	^2^Esteban et al., 2014

*Enzymes activities were measured after 30 min incubation with the inhibitor; inhibition is for the human enzyme unless specified (marked in italics). The – indicates no inhibition*.

The discovery of compounds that combine cholinesterase inhibition with binding to other targets of interest for AD is also underway. For example, the serotonin receptor, 5-HT_4_, has been linked to memory deficits (Cho and Hu, 2007; Lezoualc'h, 2007; Russo et al., 2009). Stimulation causes release of ACh and increases dopamine, serotonin, and γ-aminobutyric acid (GABA) release, and thus could act synergistically with ChE and MAO inhibition. 5-HT_4_ stimulation also increases the safer non-amyloidogenic pathway for APP cleavage (Cochet et al., 2013). Agonists or partial agonists have been designed and the first MTDLs with cholinesterase and receptor binding have been designed (Lecoutey et al., 2014; Rochais et al., 2015).

### Monoamine oxidases (MAO A, MAO B)

Neurotransmitter levels influence brain activity and preventing neurotransmitter breakdown has an anti-depressant effect. The monoamines are catabolized by MAO and COMT, inhibitors for which are useful in PD (Talati et al., 2009). Mice treated with MAOI showed significantly higher noradrenaline and serotonin levels in brain and significantly lower metabolites (including DOPAC from dopamine) (Lum and Stahl, 2012). Higher monoamine levels as a result of MAOI are also seen in rats in micro-dialysis experiments (Bazzu et al., 2013; Bolea et al., 2014), and in humans are observed as serotonin toxicity in patients given non-selective MAOI on top of serotonin reuptake inhibitors (SSRIs) (Gillman, 2011). Changes in monoamine levels also have downstream effects on expression and function of receptors and other proteins (Finberg, 2014).

Altered MAO levels are associated with brain pathology. MAO A/B knockout mice displaying anxiety-like symptoms have greatly elevated monoamine levels (Chen et al., 2004). MAO B, located mainly in glial cells, increases with age and is elevated in AD and PD (Kennedy et al., 2003; Zellner et al., 2012; Woodard et al., 2014; Ooi et al., 2015). Inhibition of MAO B by compounds in cigarette smoke is associated with delayed onset of PD, and the MAO B inhibitor, deprenyl, delays the need to begin levodopa treatment in PD patients. Considering genetic variations, the A allele of the common A644G single nucleotide polymorphism in intron 13 of the MAO B gene is associated with slightly lower platelet MAO B activity and slightly less risk of PD (Liu et al., 2014). For MAO A, a low activity allele is associated with aggression (Gallardo-Pujol et al., 2013), and the high activity that results from the long repeat allele in the promotor region of the gene is associated with depression (Meyer et al., 2006), although a Positron Emission Tomography study found no significant difference in activity MAO A activity in the human brain (Fowler et al., 2015). Inhibition of MAO A has also been shown to decrease the oxidative stress that can result from the hydrogen peroxide and the aldehyde products of MAO catalysis both in heart and brain (Kaludercic et al., 2011; Ooi et al., 2015).

#### MAO A and MAO B structure, activity, redundancy

**Figure 2:**
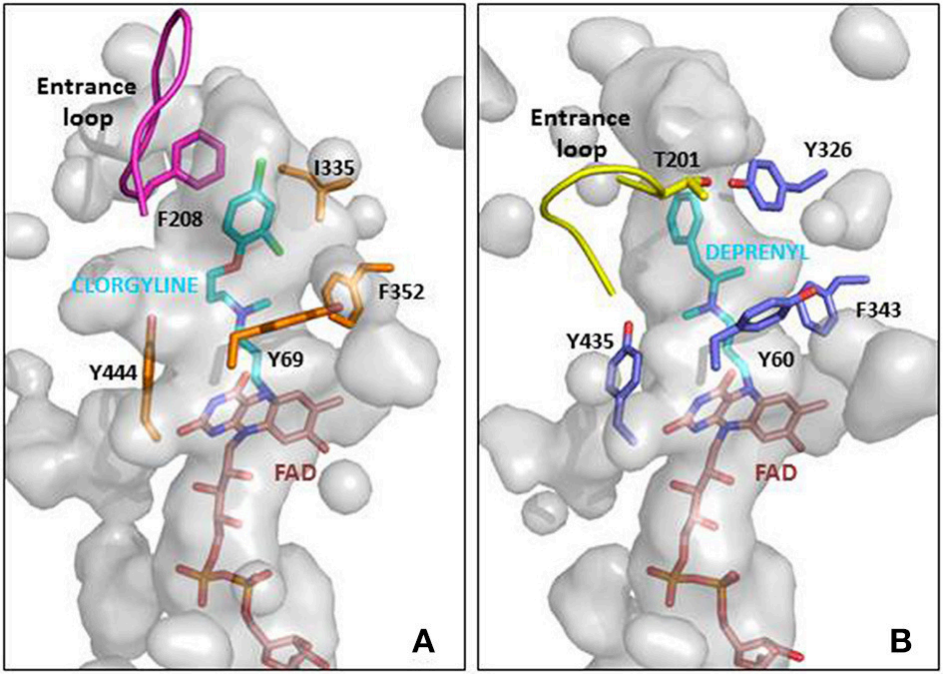
**MAO active site cavities showing the FAD cofactor and the ligand in the crystal structures**. **(A)** MAO A in complex with clorgyline (PDB ID: 2BXS) and **(B)** MAO B in complex with deprenyl (PDB ID: 2BYB). The FAD cofactors inside the cavities are shown in CPK colored sticks with carbons in pink, the clorgyline and deprenyl ligands are also shown in sticks with carbons in blue, and key residues around the ligand cavity are shown in CPK colored sticks with carbons in orange and metal blue for MAO A and MAO B, respectively. The entrance loops are highlighted in pink and yellow respectively. The PDB files were obtained from the protein databank and figures were produced using PyMOL (http://www.pymol.org).

MAO A and B share 70% homology and very similar active sites (reviewed in Edmondson et al., 2007). A major influence on substrate and inhibitor specificity is the narrow part (“gate”) of the MAO B cavity defined by I199 and Y326 (Figure 2). However, the design of selective inhibitors is not simple, although in general MAO A can accommodate bulkier compounds. Simply changing one substituent can alter affinity for one form but not the other. For example, adding a second carbonitrile group to a small furan scaffold, increased the affinity for MAO A by 10-fold but not for MAO B (Juárez-Jiménez et al., 2014) due to a hydrogen bond to asparagine 181 in MAO A. At that position (172 in MAO B) MAO B has a cysteine residue that can contribute to MAO B-selective binding. Structure-function analyses for the design of selective MAO inhibitors has been reviewed recently (Vilar et al., 2012; Patil et al., 2013; Carradori and Petzer, 2015).

Since MAO A and B are located on the X chromosome, human MAO deficiencies were first discovered in males. MAO A deficiency is associated with aggression, but MAO B deficient subjects were mentally normal. The combined deletion found in Norrie disease is associated with severe mental retardation (Brunner et al., 1993; Lenders et al., 1996). Detailed examination of the effects of deletions are now possible through knock-out mice, studies that provide insight into the roles of MAO in behavior and development (Shih and Chen, 1999; Bortolato and Shih, 2011). In mice, as in humans, MAO A deficiency is associated with aggression. MAO B deficiency does not perturb monoamine metabolism to any great extent but results in excretion of higher amounts of phenylethylamine. The substrate specificities of the two forms overlap, with MAO A metabolizing serotonin well, MAO B PEA, but both dopamine and noradrenaline. The relative efficiency of catalysis by the two forms is best expressed by the maximum catalytic velocity divided by the *K*_M_, values; these can be found in (Youdim et al., 2006). In contrast to acetylcholine neurotransmission, the primary termination of the monoamine chemical signal is by reuptake of the monoamines, first into the neuron and then back into the storage vesicles. Inhibition of MAO increases stores of monoamines, for example in PD where inhibition of MAO B slows the breakdown of dopamine (Finberg, 2014).

#### MAO A/B assay and inhibitors

MAO can be assayed using absorbance or fluorescence changes, by radiolabeled product detection, by HPLC separation of the product, or by coupling the second product H_2_O_2_ to a detection system. The simplest assay is the measurement of the oxidation of kynuramine either continuously by the absorbance change at 314 nm (Weissbach et al., 1960) or in a stopped assay by the fluorescence of the product.

Recombinant human MAO A and MAO B expressed in insect cell membranes is now commercially available but the low activity requires the sensitive coupled assay where H_2_O_2_ is used by horseradish peroxidase to convert the non-fluorescent dye, N-acetyl-3,7-dihydroxyphenoxazine (Amplex Red), to the fluorescent resorufin (Zhou et al., 1997). As with all coupled assays, considerable care must be taken to check the validity of the assay by ensuring that the enzyme of interest (MAO in this case) is rate limiting. Inhibitors can quench or enhance fluorescence, or may inhibit horseradish peroxidase. These interfering factors must be checked for each type of inhibitor. It should be noted that Amplex Red, N-acetyl-3,7-dihydroxyphenoxazine, a structure similar to the MAO A inhibitor Methylene Blue (Ramsay et al., 2007; Milczek et al., 2011) inhibits MAO A so the dye must be used at 20–50 μM, and not the 200 μM recommend by the assay kit manufacturer. Most substrates (except dopamine) can be used in this continuous coupled assay. The most frequently used is tyramine which has a K_M_ of 127 μM with MAO A and 107 μM with MAO B (Youdim et al., 2006). However, different laboratories report various values, so the K_M_ should be checked for each condition used.The discovery of highly selective reversible inhibitors for MAO A or MAO B has been the focus of compound synthesis for antidepressant design in recent years due to reduced side effects and lower drug-drug/food interaction risk. Some effective reversible inhibitors are harmine (*K*_i_ = 5 nM) (Kim et al., 1997) used to measure MAO A occupancy in positron emission tomography scans (Sacher et al., 2011) and moclobemide (used in anxiety disorders). Moclobemide, giving 70–78% occupancy of MAO A at clinically effective doses (Sacher et al., 2011), is useful because, as a reversible inhibitor, it does not inactivate the MAO A in the gut wall and so does not potentiate the vascular effects due to tyramine from the intestine. For MAO B, safinamide (*K*_i_ = 0.5 μM) (Binda et al., 2007) is in clinical trials for adjunct therapy in PD (Finberg, 2014). Traditional medicinal chemistry approaches, screening of compound libraries, and computational screening continue the search for new scaffolds for reversible inhibitors (Santana et al., 2006; Shelke et al., 2011).

However, irreversible inhibition and the slow turnover rate of MAO allows lower doses compared to reversible inhibitors and thus lower risk of side effects. All the common MAOI used clinically for depression and for PD are irreversible inhibitors (Table 1). The mechanism-based inactivation of MAO can be achieved by phenylzines, cyclopropopylamines, and propynamines. The selective irreversible inhibitors clorgyline for MAO A and deprenyl for MAO B both contain the propargyl moiety that after oxidation by MAO A forms a covalent adduct with the N5 of the FAD cofactor (Binda et al., 2002; De Colibus et al., 2005). The propargyl moiety is a useful small entity to add MAOI capability to molecules designed for other targets to give MTDL as describe below. The propargyl group must be oxidized by MAO to generate the reactive species that forms the covalent bond with the enzyme. The rate of inactivation by propargyl compounds for both MAO A and MAO B is around 0.2 min^−1^ with selectivity coming from the binding (Esteban et al., 2014; Malcomson et al., 2015). A further benefit of the propargyl moiety is its association with neuroprotection at levels lower than for inhibition of MAO (Naoi and Maruyama, 2010; Weinreb et al., 2011).

In assessing inhibitors of MAO, a final word of caution must be included regarding the considerable species differences that have been noted for inhibitor binding (Krueger et al., 1995). Happily, the human and rat sensitivities to MAOI are fairly similar but there are clear structural active site differences between the rat and human MAOs (Upadhyay et al., 2008) with implications for drug design (Novaroli et al., 2006; Fierro et al., 2007).

### Multi-target designed ligands (MTDL) that inhibit ChEs and MAOs

One promising MTDL investigated under the auspices of COST Action CM1103 is ASS234 (N-((5-(3-(1-benzylpiperidin-4-yl)propoxy)-1-methyl-1H-indol-2-yl)methyl)-N-methylprop-2- yn-1-amine). The indole group aids MAO A selectivity and the propynamine (propargyl) group allows for irreversible inhibition. However, by adding a 1-benzylpiperidine fragment (similar to the AChE inhibitor, donepezil), this compound becomes also a reversible inhibitor for AChE and BChE (Bolea et al., 2011). During biological assessment, it became apparent that this compound has neuroprotective properties, by inhibiting Aβ42 and Aβ40 self-aggregation into plaques, and by protecting against depletion of antioxidative enzymes (Bolea et al., 2013). Therefore ASS234 has been patented (PCT/ES070186; WO2011/113988 A1) as a promising compound for the treatment of AD.Many other ChE/MAO targeted MTDL have been designed in the last 5 years, either propargyl-based (Youdim, 2013; Bautista-Aguilera et al., 2014b; Samadi et al., 2015; Weinreb et al., 2015) or coumarins derivatives (Pisani et al., 2011; Patil et al., 2013; Farina et al., 2015; Xie et al., 2015). The challenge will be to add further neuroprotective properties to progress to a disease-modifying drug.

## Mitochondrial homeostasis and apoptosis

### Mitochondrial fusion, fission, and trafficking

**Figure 3:**
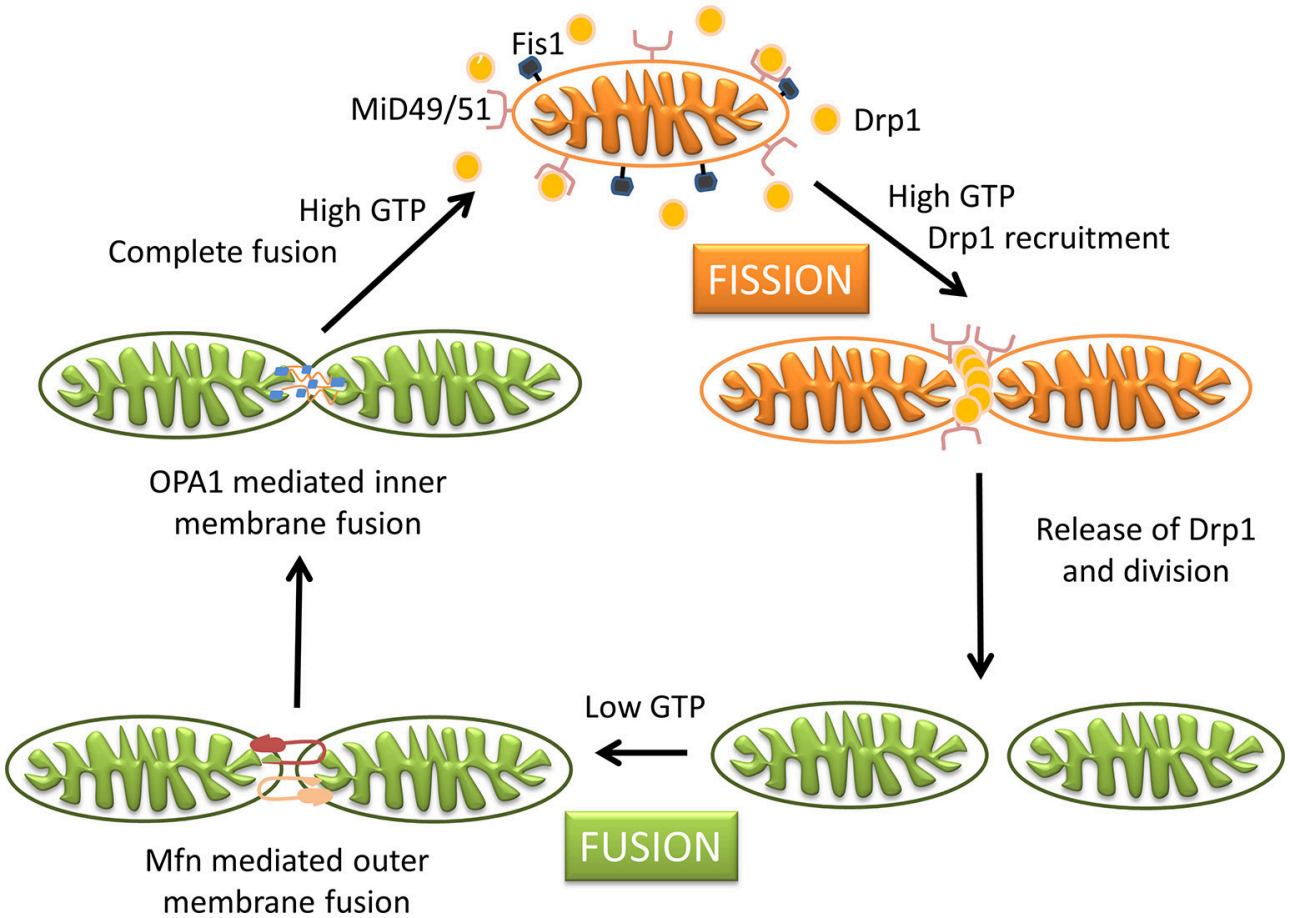
**Schematic representation of mitochondrial dynamics**. Steady state mitochondrial morphology requires a balance of fission and fusion events. During organelle fission Drp1 is recruited from the cytosol to the outer mitochondrial membrane, where it interacts directly or indirectly with Fis1 forming high molecular weight oligomers on the mitochondrial surface. This leads to constriction of mitochondria and sequential separation of the inner and outer membrane. Once Drp1 is released fission is complete. Fission also allows isolation for mitochondria that cannot be repaired followed by degradation through mitophagy, and is also important for subcellular distribution and transportation of mitochondria based on local energy needs. Mitochondrial fusion is a two-step process that requires outer and inner membrane fusion. Outer membrane fusion is facilitated by mitofusins tethering of adjacent membranes. This is subsequently followed by inner membrane fusion, which is GTP dependent and regulated by OPA1. Fusion allows for functional complementation and repair of damaged mitochondria.

Mitochondria are dynamic organelles with the ability to divide (fission) and fuse (fusion) as well as to concentrate in particular subcellular locations. Regulation of these processes is crucial for cell health and apoptosis (Hales, 2004, 2010). Fission and fusion play critical roles in maintaining functional mitochondria when cells experience metabolic or environmental stresses, a reason why their improper regulation associates with several human genetic neurodegenerative diseases affecting to neuronal survival and plasticity (Hales, 2010; Youle and van der Bliek, 2012). Fusion is proposed to mitigate stress allowing complementation by mixing contents of partially damaged mitochondria. Fission, besides being required in the creation of new mitochondria, also contributes to quality control by facilitating both removal of damaged mitochondria (mitophagy) and apoptosis under cellular stress situations (Lee et al., 2004). The combined action of several GTPases contributes to the dynamic mitochondrial networks; Drp1/Dnm1 is key in mitochondrial division, mitofusins (Mfn1 and Mfn2) control outer mitochondrial membrane fusion, and OPA1 mediates inner mitochondrial membrane fusion (Griparic et al., 2004, 2007; Ishihara et al., 2006; Cohen et al., 2008). Neurons are more sensitive than other cells to mutations in the genes coding for these proteins, indicating the importance of mitochondrial dynamics for the maintenance of the nervous system integrity (Mandemakers et al., 2007). Deletion of either of the two mitofusins results in unbalanced fission and mitochondrial fragmentation (Koshiba et al., 2004). Mutations in Mfn2 cause the Charcot-Marie-Tooth disease (Züchner et al., 2004), and mutations in OPA1 are associated with genetic forms of blindness (Delettre et al., 2000) (Figure 3). A number of other factors contribute to modulate these GTPases activities and changes in their molecular shapes precisely control these processes (Mandemakers et al., 2007). For example, several brain neurodegenerative disorders cause decrease in mitochondrial size and increased Drp1 translocation to mitochondria, increasing fission events. Treatments inhibiting Drp1 have been shown to restore mitochondrial length, reduce loss of new-born hippocampal neurons, and improve hippocampal-dependent learning and memory after damage (Li et al., 2015; Fischer et al., 2016). Therefore, reducing mitochondrial fission may contribute to rescue from brain injury, and the possibility to regulate the mechanisms of fusion and fission by different mediators in different tissues can represent a potential therapeutic target for related disorders.

Due to their complex structural and molecular features, neurons also require mechanisms for mitochondria trafficking to their distal destinations (presynaptic bouton, axons, synaptic terminals) and anchoring in regions where metabolism is in high demand. Failure to deliver a functional mitochondrion to the appropriate site within a neuron could contribute to neuronal dysfunction. Besides mitochondrial dynamics, the proteins mentioned above are also involved in mitochondrial subcellular positioning in neurons, ensuring a relatively constant mitochondrial population. As an example, membrane bound OPA1 influences mitochondrial elongation and transport in a Mfn1 dependent manner, while its soluble form regulates the tightness of mitochondrial cristae junctions and, therefore, release of apoptotic factors (Frezza et al., 2006) as well as cristae shape, which in turn, conditions supercomplex assembly (Cogliati et al., 2013). Mitochondria trafficking and anchoring mechanisms also rely on molecular motors (as KIF5 and dynein motors) which recruit mitochondria into stationary pools (Rintoul and Reynolds, 2010; Sheng and Cai, 2012; Sheng, 2014), and ensure neuronal mitochondria are adequately distributed where constant energy supply is crucial. Malfunctioning mitochondria are removed by mitophagy to minimize oxidative damage to the cell, with neurons again facing the challenge of their mitochondria being involved in distal processes located far from the cell body where lysosomes are abundant. The presence of functional lysosomes in axons has been evidenced to contribute to mitophagy of damaged mitochondria, and the local PINK1–Parkin-mediated mitophagy pathway provides rapid neuroprotection against oxidative stress without a requirement for retrograde transport to the soma (Ashrafi et al., 2014).

An imbalance of these processes involved in mitochondria dynamics and homeostasis (fission, fusion, trafficking, and mitophagy) can be detrimental to mitochondrial function, causing decreased respiration, ROS production, and apoptosis. All these are also symptoms caused by a traumatic brain injury, further indicating a prominent role of mitochondria in neuropathophysiology.

### Mutations in mitochondrial proteins

Mutations in other proteins with primary mitochondrial localization that cause abnormalities of protein conformation (mis-folding or aggregation) also result in neurodegenerative disorders. Examples include the kinase PANK2 involved in coenzyme A biosynthesis and degradation of some neurotransmitters, frataxin implicated in iron metabolism, PINK1 critical to prevent oxidative stress, or pitrilysin metallopeptidase which digests oligopeptides, including the mitochondrial fraction of amyloid-beta (Mandemakers et al., 2007; Brunetti et al., 2015). In addition, mutations in some non-mitochondrial proteins appear to affect mitochondrial function in neurodegeneration (such as superoxide dismutase 1, Parkin, α-synuclein, MAO or the kinase LRRK2), although in general the role of most of these proteins in neurodegeneration must still be elucidated (Nakamura et al., 2011; Schapira and Gegg, 2011; Haddad and Nakamura, 2015).

### Apoptosis

Apoptosis is a common type of cell death in neurodegenerative diseases, in which mitochondria make a major contribution to initiation of the death cascade (Petit et al., 1996; Naoi et al., 2006). Fission and fusion rates precisely regulate the number and morphology of mitochondria within a cell, with network fragmentation and cristae remodeling occurring during the early stages of apoptotic cell death (Wang and Youle, 2009; Youle and van der Bliek, 2012). In this context it is not surprising that proteins involved in mitochondrial morphology control also participate in apoptosis, and proteins associated with apoptosis regulation affect mitochondrial ultrastructure. Key apoptotic events in mitochondria include the release of caspase-dependent activators (cytochrome c) and caspase-independent apoptotic factors (the flavoenzyme apoptosis inducing factor, AIF), changes in electron transport, loss of mitochondrial transmembrane potential, altered cellular oxidation-reduction, and participation of pro- and anti- apoptotic Bcl-2 family proteins (Saraste and Pulkki, 2000; Edinger and Thompson, 2004). The different signals that converge on mitochondria to trigger or inhibit these events and their downstream effects delineate several major pathways in cell death (Wang and Youle, 2009).

As an example, AIF is an apoptotic factor that when released from the mitochondria and translocated to nucleus induces chromatin condensation and DNA fragmentation, while also having a vital role in mitochondria healthy cells (Susin et al., 1999; Miramar et al., 2001). Complex I (CI) dysfunction has long been associated with PD. AIF deficiency produces reduced levels of CI subunits, decreased CI activity, and impaired CI-dependent mitochondrial respiration (Vahsen et al., 2004; Urbano et al., 2005; Cheung et al., 2006). Although these AIF linked CI structural alterations have not been shown to cause dopaminergic neurodegeneration, an increase is the susceptibility of these neurons to exogenous PD neurotoxins has been proven (Perier et al., 2010, 2012). The exact role of AIF in intermembrane space of mitochondria of healthy cells has remained a conundrum, but several interesting novelties have been presented in the recent years regarding its redox activity in this organelle (Sevrioukova, 2009, 2011; Ferreira et al., 2014; Villanueva et al., 2015). Recently, it has also been described that the physical and functional NADH-dependent interaction between AIF and the protein CHCHD4 regulates the correct assembly and maintenance of the respiratory chain complexes (Hangen et al., 2015; Meyer et al., 2015). CHCHD4 participates in mitochondrial protein import and catalyzes oxidative protein folding in cooperation with the sulfhydryl oxidase GFER/ALR/Erv1p (Chacinska et al., 2008; Banci et al., 2009; Fischer et al., 2013; Koch and Schmid, 2014). Upon interaction with NADH, AIF undergoes reduction, with the concomitant dimerization and formation of highly stable charge transfer complexes. Both AIF dimers and charge transfer complexes are proposed to have a physiological function in a model where AIF would act as a sensor of the mitochondrial redox state (Churbanova and Sevrioukova, 2008; Ferreira et al., 2014; Sorrentino et al., 2015). In addition to the interplay with CHCHD4, AIF might also interact at the mitochondria with other proteins yet to be discovered.

Neurons are the cells that suffer larger effects upon deficiency of AIF, probably due to their high energetic dependency on the mitochondrial OXPHOS metabolism. In addition to AIF deficiency being related to different neurodegeneration types (Klein et al., 2002; Joza et al., 2005; van Empel et al., 2005; Cheung et al., 2006; Ishimura et al., 2008), six AIF pathological mutations have also been reported to produce human neurodegenerative diseases, with all patients with AIF mutations showing muscular atrophy, neuropathy and ataxia (Ghezzi et al., 2010; Berger et al., 2011; Rinaldi et al., 2012; Ardissone et al., 2015; Diodato et al., 2015; Kettwig et al., 2015). Thus, AIF appears as an essential protein for post-mitotic neuron survival, cerebellar development, and therefore, neurogenesis (Ishimura et al., 2008). AIF is also one of the proteins described to associate with OPA1 to cooperatively regulate and stabilize the respiratory chain, this interaction being proposed as one of the factors defining mitochondrial morphology (Cheung et al., 2006; Zanna et al., 2008).

**Figure 4:**
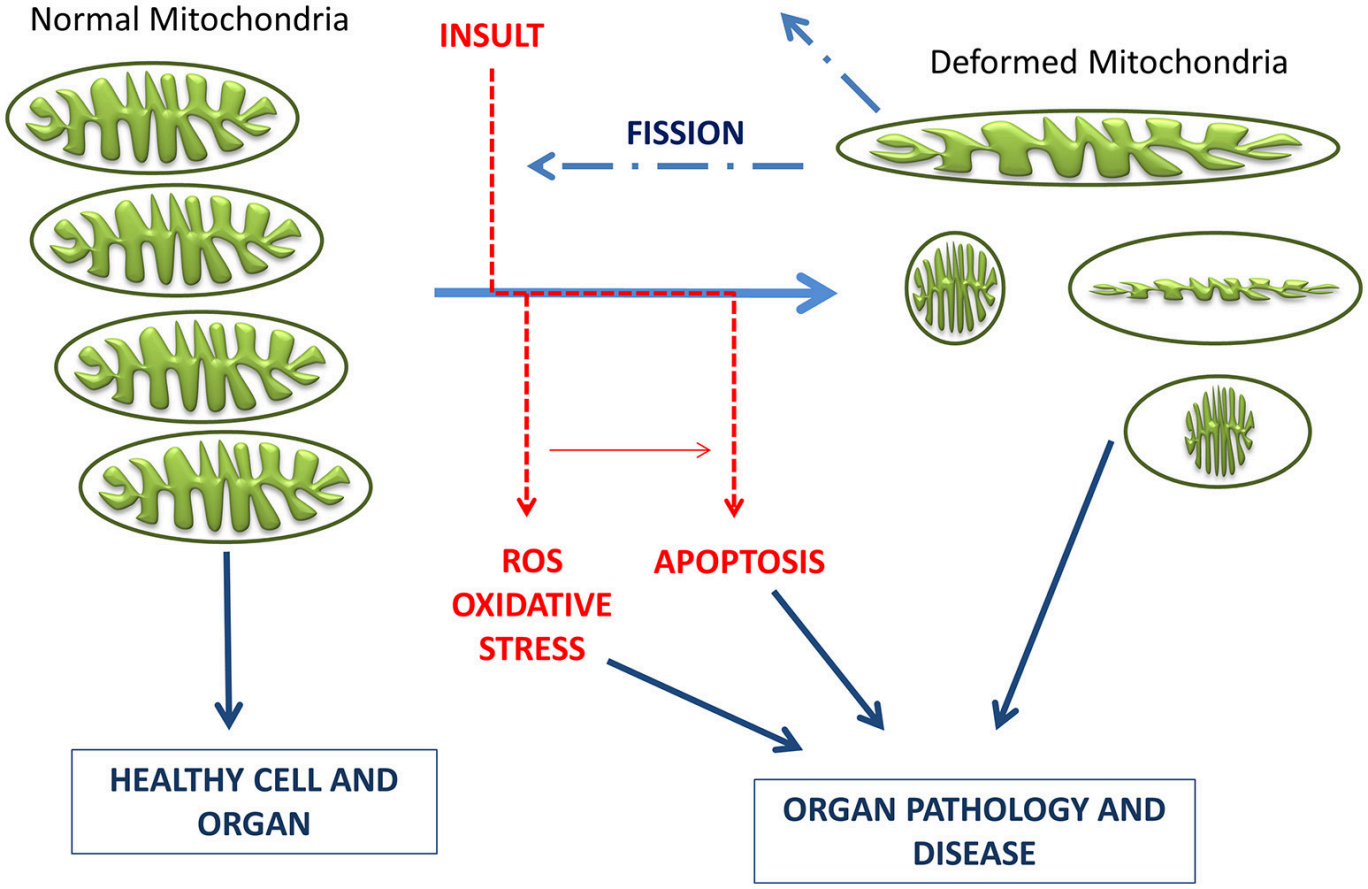
**Schematic representation of the timeline of mitochondrial bioenergetics and morphological changes inducing pathologies**. Electrons leaking from the electron transport chain generate ROS, which damage mitochondrial membrane, mitochondrial DNA, and proteins. Neurons have limited defense against oxidative damage and are highly vulnerable to ROS. Damaged/depolarized mitochondria release cytochrome c that triggers cell death by activating caspases as well as AIF that initiates apoptosis in a caspase independent manner.

The present therapeutics for neurodegenerative diseases are in general symptomatic and lack neuroprotective and neurorestorative properties, being not able to delay disease or modify its neuronal activity. In recent years, the development of multi-target neuroprotective and neurorestorative drugs with simultaneous action on enzymes such as cholinesterase, BChE and MAO A/B activities or being able to enhance the action of proteins intimately associated with mitochondrial biogenesis (Youdim, 2013; Youdim and Oh, 2013). A potential addition for this therapeutic strategy in neurodegenerative diseases is to halt common and progressive pathways for neural injury and cell death. In the current development of neuroprotective drugs, mitochondria are a key target to protect against cell death by preventing mitochondrial permeabilization, Ca^2+^ efflux, membrane potential decline and release of apoptotic factors while also inducing anti-apoptotic pro-survival proteins (Naoi et al., 2007; Weinreb et al., 2012, 2016, 2015). Connections between morphological regulation and the bioenergetics status of mitochondria are reciprocally responsive processes (Figure 4), with functional abnormality invoking morphological alterations in many human diseases and genetic defects in mitochondrial fusion/fission genes or insults inducing mitochondrial deformation (accompanied by oxidative stress and/or apoptosis) causing human diseases of lethal consequence (Galloway et al., 2012, 2014; Westermann, 2012). In this context, controlling the mitochondrial morphology by manipulating fission and fusion emerges as a future therapeutic strategy to decrease the pathological outcome.

## Antioxidant properties in an example MTDL

**Figure 5:**
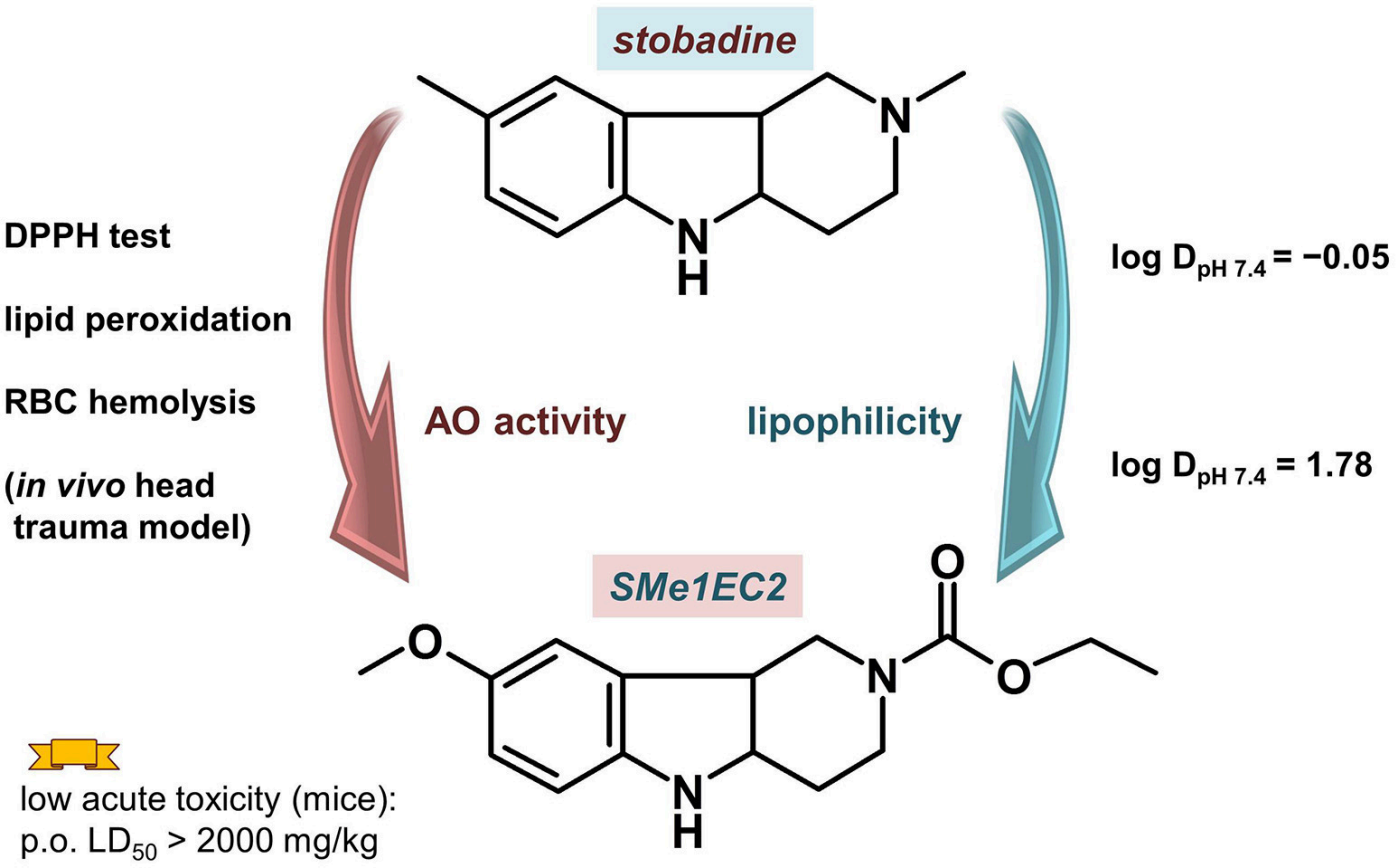
**Compound SMe1EC2 compared with the parent drug stobadine according to the structural, and ***in vitro*** and ***in vivo*** properties**.

Antioxidant properties, part of a desired pharmacologic profile for MTDLs designed to treat neurodegeneration, are screened by various *in silico, in vitro* and *in vivo* methods. Arising from the structure of the parent antioxidant drug stobadine (Horáková et al., 1994; Horáková and Stolc, 1998), several dozen derivatives with a hexahydropyridoindolic scaffold were synthesized and tested for their antioxidant and neuroprotective effect (Rackova et al., 2006; Stolc et al., 2006, 2010; Juranek et al., 2010). The aim of the new design was to use a wide knowledge of the pharmacological actions of stobadine to develop new substances with even higher antioxidant activity and reduced side effects. The screening confirmed the enhancement of the intrinsic radical scavenging activity of the 8-methoxy substituted derivatives, which was predicted for the right position of the electron-donating methoxy group. Several alkoxy-carbonyl substituents at the N2 position were tested to find sufficiently high lipophilicity and lower basicity of the molecule. From the compounds synthesized and tested (±)-cis-8-methoxy-2,3,4,4a,5,9b-hexahydro-1H-pyrido[4,3-b]indole-2-carboxylic acid ethyl ester (SMe1EC2, Figure 5), which showed enhanced antioxidant properties near a lipophilic phase, was chosen for a detailed study.

SMe1EC2 had high intrinsic scavenging activity as measured with 1,1TM-diphenyl-2-picrylhydrazyl (DPPH) (Stefek et al., 2013). The initial velocity of DPPH decolorization by 50 μM SMe1EC2 (0.507 ± 0.003 optical density(OD)/min) was comparable with that of equimolar trolox (0.494 ± 0.009 OD/min). The parent compound stobadine at 50 μM concentration was about three times less efficient (0.156 ± 0.019 OD/min). The high intrinsic activity together with enhanced lipophilicity resulted in significantly higher antioxidant properties in rat brain homogenate or in a cellular model (red cells, macrophage RAW 264.7 cell cultures) when compared with stobadine (Stolc et al., 2006; Stefek et al., 2013; Balcerczyk et al., 2014). In the experiment with red blood cells two types of initiators of the haemolysis were used: hydrophilic AAPH (2,2′-azobis(2-amidinopropane) hydrochloride) and lipophilic t-BuOOH. While the activity of more hydrophilic and basic stobadine surpassed that of SMe1EC2 in AAPH induced haemolysis, SMe1EC2 exceeded stobadine in red blood cells protection when lipophilic t-BuOOH was used (Stefek et al., 2013).

On a tissue level SMe1EC2 was able to recover the field action potential amplitude in CA1 region of rat hippocampal slices after 20 min reoxygenation following 6 min hypoxia to control value (100%) at a concentration of 3 μmol/l (Stolc et al., 2006). The field action potential, created by the pyramidal neurons in the CA1 region after electric stimulation of Schäffer collaterals in the CA3 region and involving excitation of glutamatergic synapses, is an appropriate model for functional status of brain. Neuroprotective effects of the compound were shown also in rat hippocampal slices attacked by Fe^2+^/ascorbic acid system (Gáspárová et al., 2010). Simultaneously, SMe1EC2 improved functional deficits and edema formation in rat hippocampus exposed to ischemia *ex vivo* after several days of oral treatment of rats (Gáspárová et al., 2009).

In order to estimate the *in vivo* neuroprotective potential of these new hexahydropyridoindoles, an experiment with acute head trauma model in mice has been performed (Stolc et al., 2006, 2010, 2011). There is a close relation between a traumatic head injury and a risk for later development of PD (Witcher et al., 2015; Xu et al., 2015). People aged 55 years and older who were treated in the hospital for traumatic brain injury were 44% more likely to develop PD over the next six years than those who sustained injuries, but not head injuries (Gardner et al., 2015). In the framework of the murine head trauma experiment the drugs were administered i.v. immediately after the trauma in single doses equimolar to 1 mg of stobadine dihydrochloride, and 1 h later the total sensomotoric score was monitored. SMe1EC2 proved to be excellent compound in improvement of a total sensomotoric score (Stolc et al., 2006, 2011), achieving the value 244.33 ± 50.20% (*p* = 0.0036 comparing to placebo) and exceeding such compounds as melatonin, stobadine and SPBN (2-sulfo-α-phenyl-N-tert-butyl-nitrone). During this experiment brain oedema was also evaluated by brain wet weight assessment and brain histology. After triple i.v. administration of 1.14 mg/kg of SMe1EC2 in 1 min, 2 h and 24 h after Acute Head Trauma, the increase in brain wet weight induced by the trauma and culminating 5 h after the insult was significantly diminished almost to the control level. The reduction of the oedema, occurring especially in glial cells, was also proved histologically. Moreover, the occurrence of subdural bleeding, meningeal bleeding and bleeding in brain chambers throughout the whole follow-up period (168 h) was significantly reduced.

The compound was also tested for cell protection properties in the framework of diabetes-related pathological processes. AD and type 2 diabetes mellitus present many common features (de la Monte and Wands, 2008; Correia et al., 2012c; Ahmad, 2013). Both diseases are connected with malfunctions in glucose metabolism and mitochondria, elevated oxidative stress and activation of pro-inflammatory cytokines. SMe1EC2 enhanced the viability of cultured HT22 neuronal cells exposed to high glucose with simultaneous attenuating of parameters of the oxidations stress (Rackova et al., 2009). The compound also protected rat pancreatic INS-1E β cell cultures against cytotoxic effects of hydrogen peroxide and inhibited profoundly the time-delayed apoptotic changes induced by the attack (Račková et al., 2011).

Besides metabolic disorders related to the high glucose plasma levels, pathologies connected with a high fat diet may also be related to neurodegeneration process (Morris et al., 2010). SMe1EC2 showed also efficiency in treating metabolic high-fat related disorders. In the rat model of hypertriglyceridemia it was shown that higher intake of cholesterol induced an increase in the number of active Na^+^/ K^+^-ATPase molecules in HTG rats, which resulted in the increased retention of sodium. A three-week treatment of animals kept on high cholesterol diet with SMe1EC2 in a dose of 10 mg kg^−1^ day^−1^ normalized the function of renal Na^+^/, K^+^-ATPase to the level comparable in HTG rats fed with the standard diet. For a comparison, fenofibrate in a dose of 100 mg kg^−1^ day^−1^ reversed the function of renal Na^+^/ K^+^-ATPase only slightly (Mézešová et al., 2012).

Further significant property of SMe1EC2 was its ability to protect endothelium under conditions of experimental diabetes of rats. It significantly decreased endothelaemia of diabetic rats and improved endothelium-dependent relaxation of arteries, slightly decreased ROS-production and increased bioavailability of nitric oxide in the aorta (Sotníková et al., 2011). Overall, the compound attenuated endothelial injury in diabetic animals. Although mechanism of this effect is still not clear, it could represent further positive effect in MTDL potential for treatment of neurodegenerative diseases.

Four ethological tests with rats (open field, elevated plus-maze, light/dark box exploration, forced swim test) were used to obtain information about anxiolytic and antidepressant activity of SMe1EC2 (Sedláčková et al., 2011). The substance was administered intraperitoneally 30 min before the tests at doses of 1, 10, and 25 mg/kg. SMe1EC2 was found to exert anxiolytic activity in elevated plus maze with no affection of locomotor activity in a dose-dependent manner. The middle dose of SMe1EC2 resulted in similar anti-anxiety effect manifested in rats as that of diazepam (dose 2.5 mg/kg). A medium anti-depressant activity was also predicted by combinatorial *in silico* methods (Majekova et al., 2013).

The acute toxicity of SMe1EC2 was assessed in mice after p.o. and i.v. administration. For p.o., it was estimated in GHS scale as 5, a compound with “comparatively low acute toxicity,” with the LD_50_ value over 2000 mg/kg. After i.v. administration, the LD_50_ of SMe1EC2 was 181.13 mg/kg (Stolc et al., 2006). The results of prenatal developmental toxicity study were similar: the compound demonstrated neither embryotoxic nor teratogenic effects on rat fetuses and no signs of maternal toxicity were found (Ujhazy et al., 2008).

Compound SMe1EC2 has been revealed to be a potential multi-target drug for neuronal diseases. Apart from its good distribution properties and high intrinsic radical scavenging activity, this is supported by the results of *in vivo* experiments on protection in the process of head trauma and diabetic damage of endothelium.

## Cytochrome P450

The cytochrome P450 (CYP) family is involved in different steps of therapy from drug efficacy and dose requirement to adverse drug reactions and direct toxicity (Zanger and Schwab, 2013). There are 18 mammalian CYP isoenzymes, which encode 57 genes in the human genome (Nebert et al., 2013). Of these CYP isoenzymes, more than 10 belong to the CYP1, 2, and 3 families and are responsible for the metabolism of more than 80% of xenobiotics and drugs used in therapy. This indicates that the CYP-dependent metabolism is one of the main factors in the regulation of drug concentration at a target level (pharmacokinetic effects) and is indeed involved in the adverse reactions of therapeutic compounds, in drug-drug interaction and their toxic effect. The low substrate specificity characterizing the CYP metabolism, is associated with the evidence of a large genetic polymorphism of some isoforms, particularly those involved in drug metabolism (i.e., CPY1A2, 2C9, 2C19, 2D6, and 3A4). Multi-allelic genetic polymorphisms, which remarkably depend on ethnicity, (Preissner et al., 2013) lead to distinct pharmacogenetic phenotypes termed as poor, extensive, and ultrarapid metabolizers. The loss of function promotes a reduced clearance with a consequent increase of plasma concentrations, while the gain of function leads to increased clearance and lower drug concentrations, resulting in increase and decrease effect of the drug, respectively, and potentially drug-related toxicity. These effects are not only related to genetic polymorphisms, but CYPs activity is regulated by chemicals and endogenous factors that can be promoted either by the induction or inhibition of some CYP activity. In the liver, most of the xenobiotic-metabolising CYPs are inducible, but one exception is CYP2D6. In general, control of protein expression can be exerted at the transcriptional mRNA, translational and posttranslational level. Posttranslational regulation has been described for CYP1A1, CYP1A2, CYP2E1 and CYP3A4 (Werlinder et al., 2001; Ingelman-Sundberg, 2004; Oesch-Bartlomowicz and Oesch, 2005; Smutny et al., 2013).

Pharmacoepigenomics is a new topic of research in the regulation of xenobiotic metabolizing enzymes. Up to now different studies indicate that DNA methylation and the numerous combinations of post-translational modifications of the histone proteins, are implicated in influencing the expression of genes whose products are engaged in drug metabolism. In addition, the increasing importance of the short regulatory miRNAs, has to be emphasized and initial studies show their involvement in regulating the expression of drug-metabolizing enzymes (Tsuchiya et al., 2006; Pan et al., 2009; Ingelman-Sundberg and Gomez, 2010).

Therefore, pharmacoepigenomics represents the future of research on drug metabolism, while the molecular mechanism of the transcriptional regulation of CYPs has been established and consolidated in several studies. Transcriptional control is of the highest importance and cytosolic receptors sensitive to the concentration of the environmental xenobiotics are crucial, namely the aryl hydrocarbon receptor (AhR), constitutive androgen receptor (CAR), the pregnane X-receptor (PXR), and peroxisome proliferator-activated receptor (PPARα). They regulate CYP forms as follows: CYP1A1, CYP1A2 and CYP2S1 (AhR), CYP2C9, CYP3A4 (PXR), CYP2B6, CYP2C9, CYP3A4 (CAR), and CYP4A family (PPARα) (Waxman, 1999; Ingelman-Sundberg, 2004).

All of these described regulatory mechanisms lead to the first of instances of interindividual variability in drug response, where a clear phenotypic consequence is evident in the population. Another aspect to take in account is the inhibition effects of CYP enzymes promoted by several drugs, chemicals, or diet components. This effect can increase systemic exposure, thereby causing severe toxic effects of the drug or of another concomitantly administered therapeutic compound that is metabolized by the same CYP(s) (Ludwig et al., 1999). The competition between chemicals for CYP activity has resulted in unpredictable pharmacokinetic interactions and can be a cause of drug–drug interactions, a major clinical problem.

### Cytochrome P450 in brain and its role in parkinson's disease

Most of these studies have been conducted in the liver which is the major organ involved in drug metabolism due to the high concentration of CYP in the endoplasmic reticulum of hepatocytes. However, the CYP families involved in xenobiotic metabolism are also expressed in extrahepatic tissues (i.e., intestine, brain, kidney). Since the expression of the majority of the isoforms appears to be very low compared the predominant expression in liver, and their role in overall total body clearance is lower, the basal expression and up-regulation in peripheral tissues can significantly affect local disposition of drugs or endogenous compounds and thus modify the pharmacological/toxicological effects or affect the distribution of xenobiotics in human body. In the brain, the overall level of CYP is ~0.5–2% of that in liver microsomes (Miksys and Tyndale, 2013) and could play a role in tissue- and/or cell-specific sensitivity to certain drugs or xenobiotics. There have been a number of suggestions that environmental toxins may play a role in the pathogenesis of neurodegenerative disorders by directly damaging neurons or through bioactivation of some toxic compounds via CYPs (Riedl et al., 1999; Shahabi et al., 2008; Miksys and Tyndale, 2009; Ferguson and Tyndale, 2011; Vaglini et al., 2013).

In this context, it is underlined that studies with divergent results are addressed toward the allele mutation of gene that encodes CYP2D6. This isozyme is involved in the metabolism of exogenous drugs and neurotoxins including 1-methyl-4-phenyl-1,2,3,6-tetrahydropyridine (MPTP, a neurotoxin that can cause selective dopaminergic neuronal damage) as well as endogenous compounds including dopamine (Payami et al., 2001). Recently Singh et al. (2014), in a study involving 70 PD patients, showed that a allelic variants of CYP2D6 and glutathione transferase1 were significantly associated with an increase in PD risk, due to a lower capability in the metabolism of neurotoxic compounds such as pesticides. This study is in agreement with the a meta-analysis performed by Lu et al. (2013) that demonstrated that an allele polymorphism of CYP2D6 increases the risk of Parkinson's disease.

On the contrary, other studies did not support an association between PD and mutations of the CYP2D6 and underline that PD is most likely the result of interactions between multiple genetic and environmental factors (Persad et al., 2003; Vilar et al., 2007; Halling et al., 2008). Whatever the cause of PD and other neurodegenerative disease, the knowledge of cytochrome P450 functions and metabolism is pivotal for its key roles in *in vivo* drug action, and why it plays a crucial function in the metabolism of toxic compounds.

### Cytochrome P450-dependent metabolism of MAO B inhibitors and ASS234

In the COST Action CM1103, a new family of multi-target molecules able to interact with AChE, as well as with MAO A and B, was synthesized by Samadi et al. (2011). These compounds bring together the benzylpiperidine and N-propargylamine moieties present in the AChE inhibitor donepezil and the MAO inhibitor PF9601N, respectively. The presence of propargyl moiety in the molecule confers particular susceptibility in terms of CYP-dependent metabolism. It is well-known that the terminal acetylenes can inhibit the CYP isoenzymes by alkylating the P450 prosthetic heme or by binding covalently to the protein with only partial loss of the catalytic activity. Sharma et al. (1996) demonstrated that both deprenyl and clorgyline are irreversible inhibitors of CYP2B1, by a mechanism-based inactivation due to the formation of a reactive intermediate based on their propargyl group. A recent study suggests that deprenyl can also inhibit CYP2B6 (Sridar et al., 2012). This isozyme is involved in the metabolism of Bupropion, an antidepressant often used to Parkinson's disease patients in conjunction with deprenyl, and its inhibition can lead to a potential drug interactions.

However, the inhibition of CYP 2B1 and 2B6 does not promote inhibition of CYP-dependent metabolism of the drug. In fact deprenyl in humans, as well as in experimental animals, is rapidly metabolized by the liver cytochrome CYP system, forming mainly desmethydeprenyl and methamphetamine (Baker et al., 1999; Dragoni et al., 2003b). These two compounds are further metabolized to amphetamine. The CYP-dependent metabolism showed a high hepatic clearance that justifies the low half-life of the drug observed *in vivo* in humans (~0.15 h) (Sridar et al., 2012).

It is important to note that both primary deprenyl metabolites can contribute to the therapeutic effect of the MAO-B inhibitor. Desmethyldeprenyl, a less potent inhibitor of MAO-B than the parent drug both *in vitro* and *in vivo*, is more efficacious in protecting dopamine neurons against oxidative stress damage (Olanow and Tatton, 1999). The other metabolite, methamphetamine, is a more potent inhibitor of presynaptic noradrenaline and dopamine uptake than the parent drug and it has been suggested that this effect contributes to neuroprotection (Sziráki et al., 1994).

These metabolic pathways are also active in the CNS, as observed *in vitro* in microsomal preparations of monkey and mouse brain (Dragoni et al., 2003a). In contrast to deprenyl, PF9601N, the precursor of MTDL compounds studied in the COST CM 1103 project (Bolea et al., 2011), showed significantly lower liver clearance. The *in vivo* treatment of C57BL/6 mice did not modify cytochrome P450 and b5 content, and did not change NADPH-CYP-reductase or CYP2E1, 2A5, 1A1, 2B6, 3A activities. Furthermore, CYP-dependent metabolism of PF9601N by liver microsomes from either control or treated mice gave rise only to the formation of the desmethyl metabolite, FA72 (Dragoni et al., 2007). This desmethyl compound promoted a concentration-dependent inhibition of peroxinitrite oxidation with an IC_50_ value lower than the parent compound and than deprenyl. Furthermore, PF9601N and its metabolite were able to strongly inhibit rat brain neuronal nitric oxide synthase, (NOS) in contrast to observations with deprenyl, which caused a slight decrease of the enzyme activity only at millimolar concentration (Bellik et al., 2010).

These observations led us to study the CYP-dependent metabolism of ASS234 (Marco-Contelles et al., 2016). ASS234 was incubated in phosphate buffer with human or rat hepatic microsomal preparations (HLM and RLM, respectively) as previously reported (D'Elia et al., 2009). Samples were analyzed by Agilent UHD Accurate-Mass Q-TOF LC/MS and the experimental data obtained were elaborated using Mass-MetaSite, a computer assisted method for the interpretation of LC–MSMS data that combines prediction of a compound's site of metabolism (SoM) with the processing of MS spectra and rationalization based on fragment analysis (Strano-Rossi et al., 2014). The kinetic analysis indicated that the substrate depletion followed a mono-exponential relationship either in presence of HLM and RLM. RLM metabolized the compound at a higher rate compared to HLM. In fact, after 30 min incubation only 23% of ASS234 was metabolized by human preparations, while RLM preparations were able to metabolized more than the 80% of initial amount (10 μM) of substrate (Simone et al., 2014).

**Figure 6:**
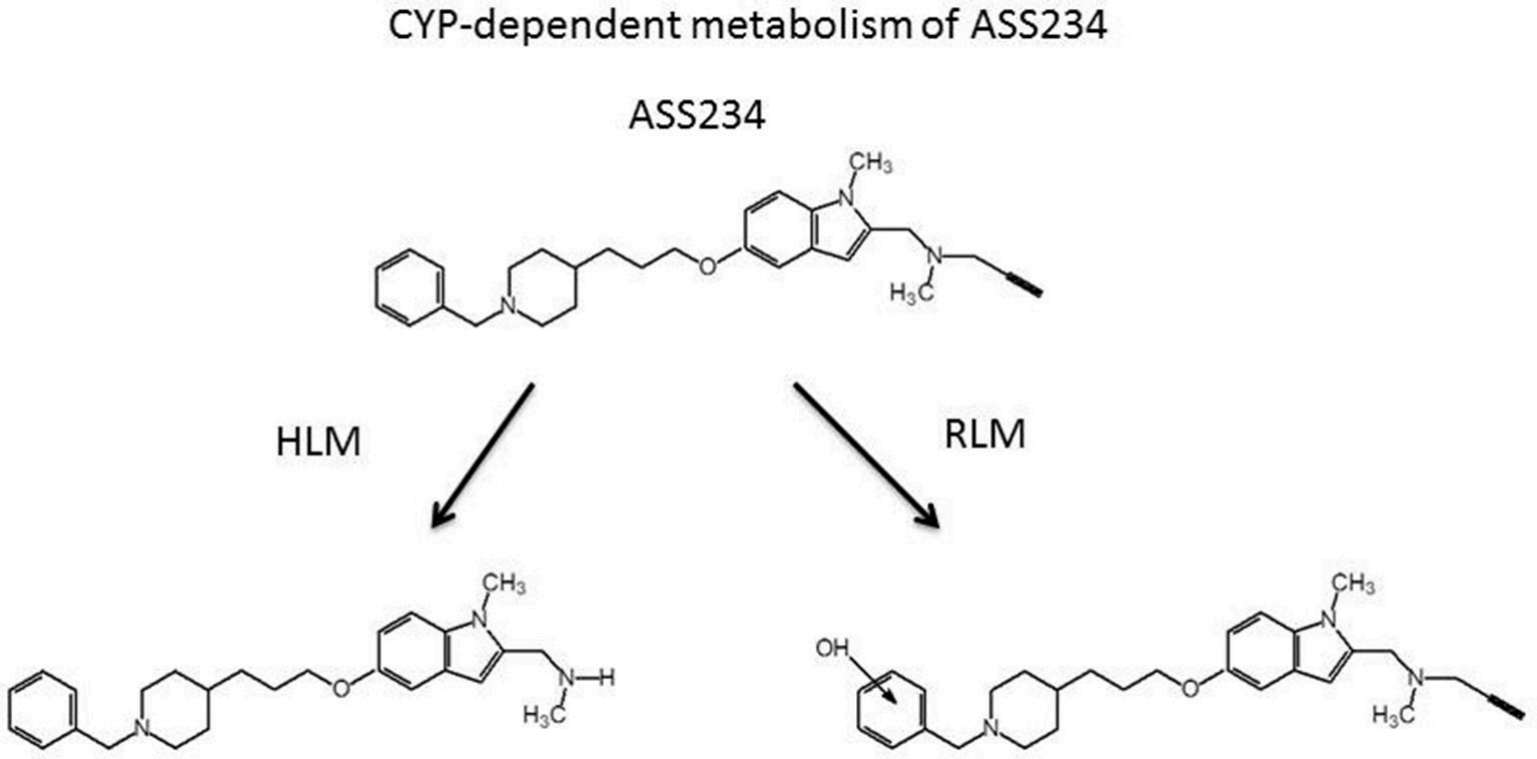
**Cytochrome P450-dependent metabolism of ASS234 in human (HLM) and rat (RLM) liver microsomal preparations**. ASS234 (25 μM) was incubated at 37°C in phosphate buffer in the presence of microsomes for 30 min.

The MS analysis of the products from ASS234 metabolism showed two different pathways as shown in Figure 6. The principal metabolite observed with HLM resulted in a compound at [M-38]^+^ (m/z) indicating the formation of N-depropargylated metabolite, in agreement with that observed for the CYP-dependent metabolism of PF9601N (Dragoni et al., 2007). On the contrary, in RLM preparations, the major metabolite resulted in m/z equal to [M +16]^+^, which corresponded to the hydroxylated derivative on the benzene ring. Other minor peaks were present in both microsomal preparations and resulted in, as secondary metabolites, the N-demethylated derivatives either on tertiary amine or indole nitrogen. The *in silico* analysis indicated that CYP2D6 and 2C19 are the major CYPs involved in the human metabolism of ASS234 (Simone et al., 2014).

Taken together, this information clearly indicates that ASS234 is a poor CYP(s) substrate in human liver, that the resulting metabolite should be not a MAO inhibitor, but that the inhibition effect on AChE should remain. Furthermore, in accord with the observations with PF9601N (Dragoni et al., 2007), the ASS234 CYP-dependent metabolite can be a more potent antioxidant and NOS inhibitor than the parent compound.

However, the involvement of CYP2D6 and 2C19, two highly genetic polymorphic cytochrome P450s, require more care due to possible toxic effects of the parent compound having a lower metabolic clearance. Moreover, the evidence that human and rat present two different metabolic behaviors, in terms of velocity and metabolite formation, underlines the differences between species in CYP-dependent metabolism and the danger of attempting to extrapolate results across species.

## Conclusion

In the last century pharmacological research was driven to discover highly selective drugs. This strategy has failed, in part, because it is seen that the interaction with a single target, either receptor or enzyme, can promote compensatory adaptations in the living organisms leading to a failure of the therapy. These observations and the discovery that different pathologies have common aspects has led to the synthesis of new molecules that can interact with multiple targets with the aim to improved balance of efficacy and safety compared to single targeting drugs.

We have reviewed the major targets for the assessment of MTDL relevant to neurodegenerative diseases, giving examples of compounds generated by our collaborating medicinal chemists in COST Action CM1103. Mitochondria are highlighted as the area of future interest but the many possible targets will have to be refined to those most influential on progression in each specific disease. It is becoming recognized, particularly for mitochondrial function, that the cumulative effect of small inefficiencies can trigger pathology under additional insult such as oxidative stress. Recent advances in cell biology techniques have enabled the study of factors involved in mitochondrial dynamics. The processes vital to mitochondrial health are also vital to neuronal survival and will provide the challenge to discover new tools to prevent neurodegeneration. When single target efficacy is achieved, then new modalities can be added to MTDL for the ultimate prevention of neuropathology.

## Author contributions

All authors contributed to the writing of the manuscript. MV and RR integrated and revised it.

### Conflict of interest statement

The authors declare that the research was conducted in the absence of any commercial or financial relationships that could be construed as a potential conflict of interest.

## Abbreviations

AD: Alzheimer's Disease
AChE: acetylcholinesterase
Aβ: amyloid beta
BChE: butyrylcholinesterase
MAO: monoamine oxidase
CYP: cytochrome P450
AIF: apoptosis inducing factor
MTDL: multi-target designed ligand
PD: Parkinson's Disease
ROS: Reactive Oxygen Species
CI: Complex I
HLM: human liver mitochondria
RLM: rat liver mitochondria.
